# The *Klebsiella pneumoniae* tellurium resistance gene *terC* contributes to both tellurite and zinc resistance

**DOI:** 10.1128/spectrum.02634-24

**Published:** 2025-04-09

**Authors:** Ruixiang Yang, Shuang Han, Yanshuang Yu, Hongru Li, John D. Helmann, Katharina Schaufler, Michael D. L. Johnson, Qiu E. Yang, Christopher Rensing

**Affiliations:** 1 Institute of Environmental Microbiology, College of Resources and Environment, Fujian Agriculture and Forestry University 602381, Fuzhou, Fujian, China; 2 Fujian Provincial Key Laboratory of Soil Environmental Health and Regulation, College of Resources and Environment, Fujian Agriculture and Forestry University 602381, Fuzhou, Fujian, China; 3 Fujian Provincial Key Laboratory of Medical Big Data Engineering, Fujian Provincial Hospital, Shengli Clinical College of Fujian Medical University 117861, Fuzhou, Fujian, China; 4 Department of Respiratory and Critical Care Medicine, Fujian Shengli Medical College, Fujian Provincial Hospital, Fujian Medical University 74551, Fuzhou, Fujian, China; 5 Department of Microbiology, Cornell University 5922, Ithaca, New York, USA; 6 Department of Epidemiology and Ecology of Antimicrobial Resistance, Helmholtz Centre for Infection Research HZI, Helmholtz Institute for One Health 657809 , Greifswald, Mecklenburg-Vorpommern, Germany; 7 Department of Immunobiology, The University of Arizona College of Medicine Tucson 12216 , Tucson, Arizona, USA; JMI Laboratories, North Liberty, Iowa, USA

**Keywords:** *Klebsiella pneumoniae*, TerC, zinc detoxification, virulence

## Abstract

*Klebsiella pneumoniae* is widely recognized as a pathogen responsible for hospital-acquired infections and community-acquired invasive infections. It has rapidly become a significant global public health threat due to the emergence of hypervirulent and multidrug-resistant strains, which have increased the challenges associated with treating life-threatening infections. Tellurium resistance genes are widespread on virulence plasmids in *K. pneumoniae* isolates. However, the core function of the *ter* operon (*terZABCDEF*) in *K. pneumoniae* remains unclear. In this study, the multidrug-resistant *K. pneumoniae* P1927 strain was isolated from the sputum of a hospitalized pneumonia patient. The *ter* operon, along with antimicrobial resistance and virulence genes, was identified on a large hybrid plasmid in *K. pneumoniae* P1927. We generated a *terC* deletion mutant and demonstrated that this mutant exhibited reduced virulence in a *Galleria mellonella* larva infection model. Further physiological functional analysis revealed that *terC* is not only important for Te(IV) resistance but also for resistance to Zn(II), Mn(II), and phage infection. All genes of the *ter* operon were highly inducible by Zn(II), which is a stronger inducer than Te(IV), and the *terBCDE* genes were also induced by Mn(II). Collectively, our study demonstrates novel physiological functions of TerC in Zn(II) resistance and virulence in *K. pneumoniae*.

**IMPORTANCE:**

*Klebsiella pneumoniae* has rapidly become a global threat to public health. Although the *ter* operon is widely identified in clinical isolates, its physiological function remains unclear. It has been proposed that proteins encoded by the *ter* operon form a multi-site metal-binding complex, but its exact function is still unknown. TerC, a central component of the tellurium resistance determinant, was previously shown to interact with outer membrane proteins OmpA and KpsD in *Escherichia coli*, suggesting potential changes in outer membrane structure and properties. Here, we report that TerC confers resistance to Zn(II), Mn(II), and phage infection, and Zn(II) was shown to be a strong inducer of the *ter* operon. Furthermore, TerC was identified as a novel virulence factor. Taken together, our results expand our understanding of the physiological functions encoded by the *ter* operon and its role in the virulence of *K. pneumoniae*, providing deeper insights into the link between heavy metal(loid) resistance determinants and virulence in pathogenic bacteria.

## INTRODUCTION

Gram-negative *Klebsiella pneumoniae* is a significant global public health threat, causing opportunistic healthcare-associated infections and severe community-acquired infections (1). Recently, this pathogen has evolved hypervirulent and multidrug-resistant traits through the horizontal acquisition of various extended-spectrum beta-lactamases (ESBLs) genes, carbapenemase genes, and virulence genes (2–4). Many *K. pneumoniae* isolates typically carry large hybrid virulence plasmids, which encode numerous antimicrobial resistance (AMR) determinants as well as virulence factors that contribute to their ability to cause disease in humans (5–8). In addition, some of these plasmids also carry heavy metal(loid) resistance (HMR) genes, conferring resistance to copper (*pcoABCDERS*), lead (*pbrABCR*), mercury (*merACDEPT*), silver (*silCERS*), and tellurium (*terZABCDEF*) (9–11).

The *ter* operon was first identified on plasmid pMER610, which was shown to be responsible for converting tellurite into a black metallic tellurium element, deposited within the inner membrane and the intracellular periplasmic space (12, 13). However, tellurium is an extremely rare and toxic metalloid that both bacterial species and humans seldom encounter (14, 15). Interestingly, the *ter* operon is widespread among bacterial pathogens, including *Escherichia coli* O157:H7 (16), *Listeria monocytogenes* (17), *Staphylococcus aureus* (18), *Enterococcus faecais* (19), and *K. pneumoniae* (20). In *Proteus mirabilis*, the *ter* operon was found to be inducible not only by tellurite but also, to a lesser extent, by oxidative stress (21). This suggests that the *ter* operon may provide bacterial pathogens with a selective advantage beyond high-level tellurium resistance (22). Previous efforts to identify the primary physiological function of the *ter* operon revealed that transpositions into *terD*, *terC*, and *terZ* reduced or abolished phage inhibition, oxidative stress resistance, and colicin resistance phenotypes. In contrast, insertions into *terE* and *terF* had no effect on these phenotypes, whereas insertions in *terA* only reduced phage inhibition levels (23). Furthermore, the *ter* gene products and TelA are the center of membrane-linked metal recognition complexes and were shown to regulate phosphorylation-dependent signal transduction, RNA-dependent regulation, biosynthesis of nucleoside-like metabolites, and DNA processing (24). More importantly, the presence of the *ter* operon enhances the overall fitness and survival of the bacterial strain during macrophage attacks (25). Similarly, the *terZAB* in *Yersinia pestis* has been proposed to mediate a filamentous response during macrophage infections, possibly serving as a bacterial adaptive strategy to counteract macrophage-associated stresses (26).

Many *K. pneumoniae* isolates carrying the *ter* operon have been isolated from neonatal sepsis cases (London, England), dairy cattle mastitis milk (11 states, USA), patient cohort, and fecal samples from healthy volunteers (Fujian, China) (9, 27–29). The *ter* operon is typically located on a large plasmid and is genetically independent of other plasmid-encoded virulence and antibiotic resistance loci (9, 28). Moreover, the *ter* operon is strongly associated with hypervirulent clonal groups CG23, CG65, and CG86. It has also been identified on a conjugative plasmid harboring key virulence genes (*rmpA*, *iroBCDN*, *rmpA2,* and *incABCD-iutA*), which exhibits high virulence in mice (30, 31). The strong association of the *ter* operon with hypervirulence plasmids in *K. pneumoniae* highlights the need to define the true physiological function of the *ter* operon in *K. pneumoniae*. It has been demonstrated that the *ter* operon is significantly associated with *K. pneumoniae* infections based on six patient-level variables from a clinical model (28). Subsequent research using multiple mouse models of infection and colonization further revealed that the *ter* operon enhances fitness in the gut (32). Additionally, a recent study determined that the *ter* protein TerC functions as a bladder fitness factor and is necessary for tolerance to ofloxacin, polymyxin B, and cetylpyridinium chloride (33). TerC family proteins are also encoded independently of *ter* operons in several bacteria species and have been studied in both *E. coli* and *B. subtilis*. In these systems, TerC facilitates the export of Mn(II) across the inner membrane and has been implicated in the co-translocational metalation of secreted proteins (34, 35). Despite these extensive studies, the core function of the *ter* operon in *K. pneumoniae* remains elusive.

In this study, an isolate, *K. pneumoniae* P1927, exhibiting a multidrug-resistant phenotype and high resistance to heavy metal(loid)s, was isolated from the sputum of a hospitalized pneumonia patient. We analyzed the whole genome sequence of P1927 and identified a plasmid encoding the *ter* operon (*terY-CYXYW-ZABCDEF*), which is highly conserved and broadly distributed among different bacteria but is especially prominent in *K. pneumoniae* isolates. To characterize the correlation between the *ter* operon and virulence in *K. pneumoniae* P1927, the most conserved gene in the *ter* operon, *terC*, was deleted. Using the *Galleria mellonella* larvae infection model, we found that deletion of *terC* reduced the virulence of *K. pneumoniae* P1927. Interestingly, we also discovered that *terC* was important not only for conferring Te(IV) resistance but also for resistance to Zn(II), Mn(II), and phage infection. Furthermore, the *ter* operon was highly inducible by Zn(II), and Zn(II) acts as a stronger inducer of the *ter* operon compared with Te(IV). Taken together, our data reveal that *terC* is associated with virulence in *K. pneumoniae* P1927 and implicated in Zn(II) resistance.

## RESULTS

### Phenotypic antimicrobial and heavy metal(loid)s resistance of *K. pneumoniae* P1927

*K. pneumoniae* strain P1927 was isolated from the sputum of a hospitalized pneumonia patient. To determine the minimum inhibitory concentrations (MICs) of antimicrobials, the agar dilution method was employed to assess susceptibility to a panel of 26 antimicrobial agents, following the Clinical and Laboratory Standards Institute (CLSI) guidelines. Strain P1927 exhibited resistance to almost all tested antimicrobials (22/26) (Table 1), showing alarming levels of resistance. Notably, strain P1927 displayed high-level resistance to ampicillin, kanamycin, and gentamycin (MIC ≥ 1,024 µg/mL). In contrast, it was sensitive to apramycin, tigecycline, rifampicin, and colistin. These MIC results indicate that strain P1927 exhibits a multidrug-resistant (MDR) phenotype, as it displays resistance to at least one agent in three or more antimicrobial classes (36).

**TABLE 1:** MIC results of *K. pneumoniae* P1927 from antimicrobial susceptibility testing^*a*^

Antibiotics	AMP	AMX	PIP	CZO	FOX	CRO	FEP	ATM	ETP	IPM	KAN	GEN	TOB
MIC (µg/mL)	≥1024	≥32	≥128	≥64	≥64	≥64	≥64	≥64	≥8	≥16	≥1024	≥1024	≥16
Antibiotics	CIP	LFX	SXT	MNO	SUL	APR	STR	TCY	CHL	TGC	FLR	RIF	COL
MIC (µg/mL)	≥4	≥8	≥320	≥14	≥6	−	≥4	≥64	≥64	−	≥16	−	−

^*a*^ Notes: −: sensitivity; AMP: ampicillin; AMX: amoxicillin; PIP: piperacillin; CZO: cephazolin; FOX: cefoxitin; CRO: ceftriaxone; FEP: cefepime; ATM: aztreonam; ETP: ertapenem; IPM: imipenem; KAN: kanamycin; GEN: gentamycin; TOB: tobramycin; CIP: ciprofloxacin; LFX: levofloxacin; SXT: cotrimoxazole; MNO: minocycline; SUL: sulbactam; APR: apramycin; STR: streptomycin; TCY: tetracycline; CHL: chloramphenicol; TGC: tigecycline; FLR: florfenicol; RIF: rifampicin; COL: colistin.

An increasing number of studies have reported that hybrid plasmids in *K. pneumoniae* isolates are strongly associated with genes encoding heavy metal(loid) resistance determinants (9, 37). Moreover, elevated resistance to heavy metal(loid)s was also detected in strain P1927. Notably, strain P1927 was not only resistant to essential nutrient metal ions, such as Cu(II), Zn(II) and Mn(II), but also to toxic metal(loid) ions, such as Pb(II), As(III), Sb(III), and Te(IV) (Table 2). This suggests that the genome of strain P1927 harbors numerous genes encoding HMR determinants.

**TABLE 2:** MIC results of *K. pneumoniae* P1927 from heavy metal(loid)s susceptibility testing

Heavy metal(loid)s	Te(IV)	Zn(II)	Mn(II)	Cu(II)	Ni(II)	Co(II)	As(III)	Ag(I)	Cd(II)	Pb(II)	Rox(V)	Sb(III)	Sb(V)
MIC (mM)	1	7	21	12	4	1	8	0.2	0.7	12	5	9	1

### Genomic features of *K. pneumoniae* P1927

**Fig 1:**
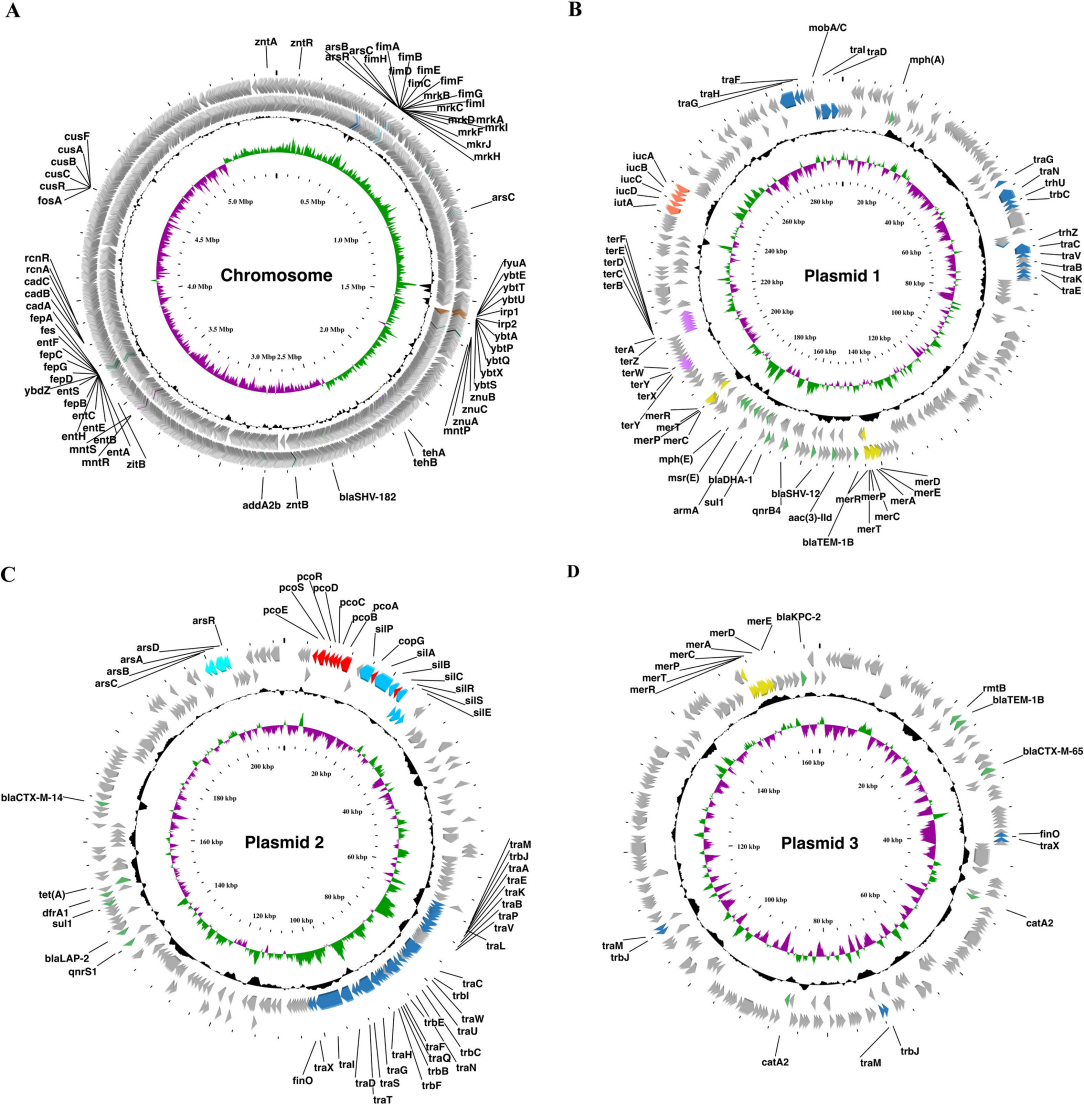
Circular genome map of *K. pneumoniae* P1927. (A) Chromosome circle map. (B) Circle map of plasmid 1. (C) Circle map of plasmid 2. (D) Circle map of plasmid 3. From the outside to the inside, the first two circles represent the coding sequences (CDS) on the forward and reverse strands. The third circle represents the GC content, with the outer part indicating that the GC content in this region was higher than the average GC content of the whole genome. The fourth circle represents the GC skew value. When the value was positive, the positive chain was more likely to transcribe the CDS, and when it was negative, the negative chain was more likely to transcribe the CDS. The circular genome map was generated by CGview (v.1.0). Antimicrobial resistance genes, heavy metal(loid) resistance genes, conjugative transfer genes, and virulence factors were labeled.

The genome of strain P1927 was determined to be 6,172,359 bp in length, with an overall GC content of 56.6%, containing one chromosome and three plasmids (Fig. 1). The complete genome contains 5,896 putative coding sequences (CDSs), 87 tRNAs, 25 rRNAs, and 13 ncRNAs. The complete genome sequence was submitted to the National Center for Biotechnology Information (NCBI) and assigned the GenBank accession numbers CP073377.1, CP073378.1, CP073379.1, and CP073380.1. Based on the genome sequence, the sequence type of strain P1927 was predicted to be ST11, and its surface capsule loci were identified as K64.

Highly diverse AMR genes were identified in the genome of strain P1927 using ResFinder and The Comprehensive Antibiotic Resistance Database (Fig. 1; Table S3). Strain P1927 was predicted and confirmed to be an extended-spectrum beta-lactamase (ESBLs) and carbapenemase producer. The ESBL-encoding genes detected in the genome were highly diverse, including *bla*_SHV-182_, *bla*_TEM-1B_, *bla*_SHV-12_, *bla*_DHA-1_, *bla*_CTX-M-14_, *bla_LAP-2_*, and *bla*_CTX-M-65_, whereas only one carbapenemase-encoding gene, *bla*_KPC-2_, was identified on plasmid 3 (Fig. 1; Table S3). Additionally, non-ESBLs resistance genes and genes conferring resistance to other classes of antimicrobials were also detected on the genome (Fig. 1; Table S3).

In addition to AMR genes, the core pathogenicity operons, *fim* (*fimHGFDCTAEB*) and *mrk* (*mrkABCDFJTH*), were also identified on the P1927 chromosome (Fig. 1A). The *fim* gene cluster encodes type 1 fimbriae, whereas the *mrk* gene cluster encodes type 3 fimbriae, which have been shown to be involved in adherence and biofilm formation, respectively (38). Two siderophore systems were also detected: yersiniabactin, synthesized by *ybtETUAPQXS* and regulated by *irp1/2*, along with its receptor encoded by *fyuA*, and the core siderophore enterobactin, synthesized by *entDFSCEBAH* and *fepACGDB*, which are essential for iron acquisition from host cells (39, 40). Based on analysis using PlasmidFinder, plasmid 1 of strain P1927 is a large hybrid virulence plasmid (~300 kb) with both IncFIB and IncHI1B replicons on the pNDM-MAR backbone (Fig. S1). This plasmid harbors the hypervirulent *K. pneumoniae* (HvKp) characteristic virulence determinant, aerobactin siderophore, synthesized by *intA* and *iucDCBA* (Fig. 1B). However, plasmid 1 lacks the mucoid regulator gene (*rmpA2*), which is present on the traditional hypervirulence plasmid pK2044 harbored by strain NTUH-K2044 (6).

Additionally, multiple HMR genes encoding resistance to zinc (*znuBCA*, *zntRAB*, and *zitB*), manganese (*mntPRS*), tellurium (*tehAB*, *terY-CYXYW-ZABCDEF*), mercury (*merEDACPTR*), arsenic (*arsCBADR*), copper (*pcoESRDCBA*, *copG*, and *cusRABCF*), nickel (*rcnRA*), cadmium (*cadABC*), and silver (*silPABCRSE*) were detected on the chromosome and plasmids of strain P1927 (Fig. 1). Previously, hypervirulent *K. pneumoniae* isolates have been shown to be strongly associated with genes encoding tellurium and other heavy metal(loid) resistance determinants (30). Two large conjugative transfer gene clusters were also identified on plasmids 1 and 2 of strain P1927, respectively (Fig. 1B and C). Taken together with phenotypic assays, these results showed that strain P1927 is an ESBL- and carbapenemase-producing multidrug-resistant hypervirulent *K. pneumoniae* isolate.

### The distribution of the *ter* operon in different bacteria

**Fig 2:**
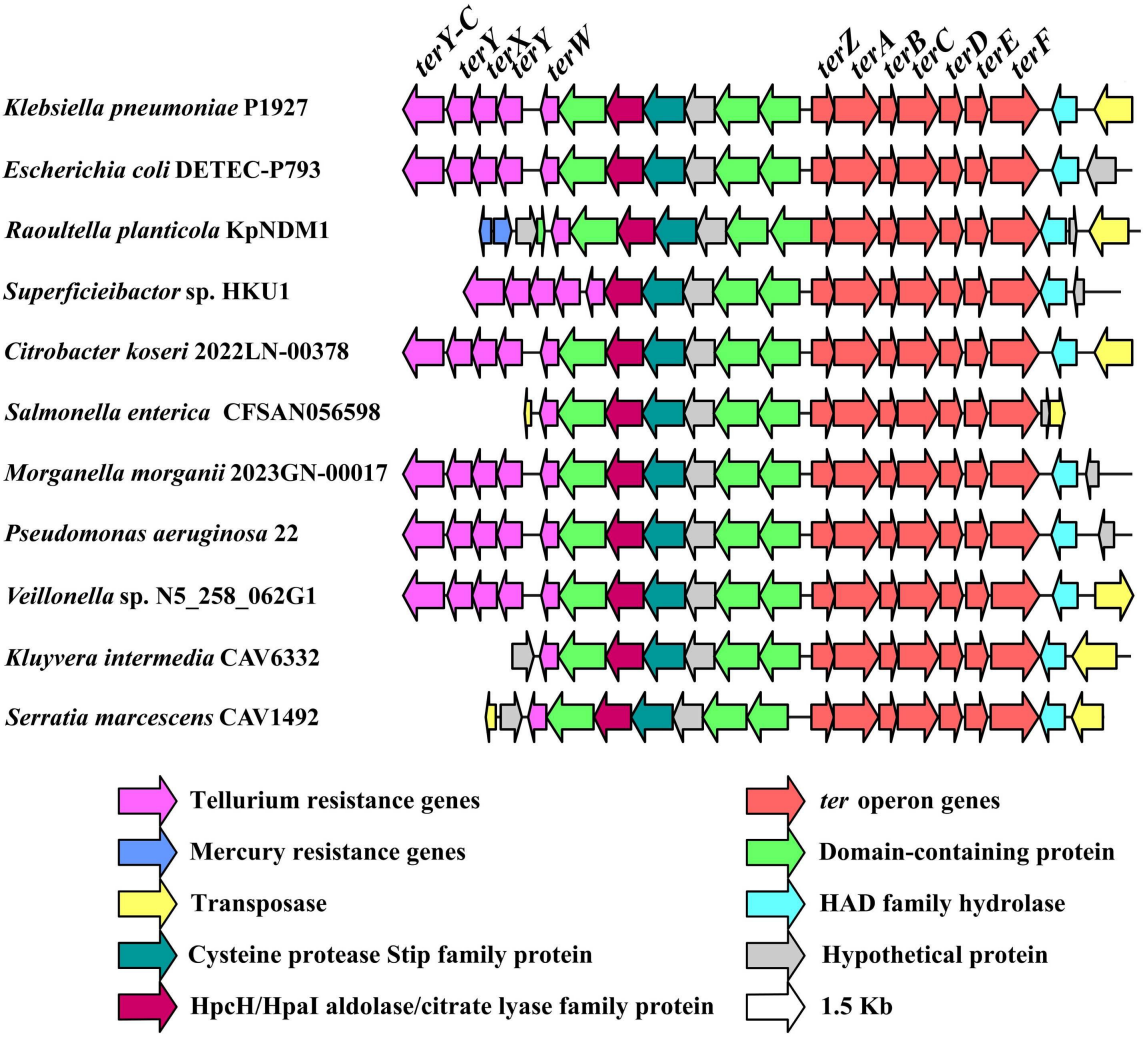
Distribution of the *ter* operon in different bacteria (GenBank accession numbers in parentheses). *K. pneumoniae* P1927 (CP073378.1), *E. coli* DETEC-P793 (CP116116.1), *R. planticola* KpNDM1 (JX515588.1), *Superficieibactor* sp. HKU1 (CP119754.1), *C. koseri* 2022LN-00378 (ABJULC000000000.3), *S. enterica* CFSAN056598 (AACWHP000000000.1), *Morganella morganii* 2023GN-00017 (ABKLBV000000000.3), *P. aeruginosa* 22 (JASEZL000000000.1), *Veillonella* sp. N5_258_062G1 (JAWFEU000000000.1), *Kluyvera intermedia* CAV6332 (DACSNU000000000.1), *Serratia marcescens* CAV1492 (CP011641.1).

To determine the distribution of the *ter* operon in other bacteria, the gene cluster containing the *ter* operon was extracted from NCBI (Fig. 2). The map was generated using Illustrator for Biological Sequences (IBS) (v.1.0) (41). Based on BLAST result from NCBI, the *ter* operon was shown to be more prevalent in *K. pneumoniae* isolates, and homologous gene clusters of the *ter* operon were frequently identified in various pathogenic bacteria isolated from patients and hospital environments, including *E. coli*, *Raoultella planticola*, *Superficieibactor* sp., *Citrobacter koseri*, *Salmonella enterica*, *Pseudomonas aeruginosa*, and *Veillonella* sp. (Fig. 2). In contrast, *K. pneumoniae* isolates were obtained from a variety of sources, including patients (blood, urine), animals (horse, chicken), and environmental samples such as fruits and wastewater. In addition, a recent study identified *K. pneumoniae* isolates carrying the *ter* operon in dairy cattle mastitis milk (27).

In addition to the *ter* operon, the *terY-CYXYW* gene cluster was also identified upstream of the *ter* operon in *E. coli*, *Superficieibactor* sp., *C. koseri*, *M. morganii*, *P. aeruginosa*, and *Veillonella* sp. (Fig. 2). However, it appears that only the *terW* gene is conserved in these isolates compared with the *ter* operon, suggesting that *terW* plays a pivotal role in the *terY-CYXYW* gene cluster. Coincidentally, it has been determined that the detection rate of *terW* in hypervirulent *K. pneumoniae* (HvKp) (*rmpA*^+^/*iutA*^+^) (70.6%) is significantly higher than that in classic *K. pneumoniae* (cKp) (*rmpA*^-^ and *iutA*^-^) (13.3%) (29). Furthermore, TerW, encoded in the vicinity of the *terZABCDE*, has been shown to regulate the expression of the *ter* operon (42). Additionally, genes encoding transposases were identified downstream of the *ter* operon (Fig. 2), suggesting that the spread of the *ter* operon among different bacteria is likely mediated by transposition. Overall, these results indicate that the *ter* operon in different bacteria shares conserved sequences and is strongly associated with infections in patients colonized by pathogenic isolates.

### TerC contributes to Te(IV), Zn(II), and Mn(II) resistance in *K. pneumoniae* P1927

**Fig 3:**
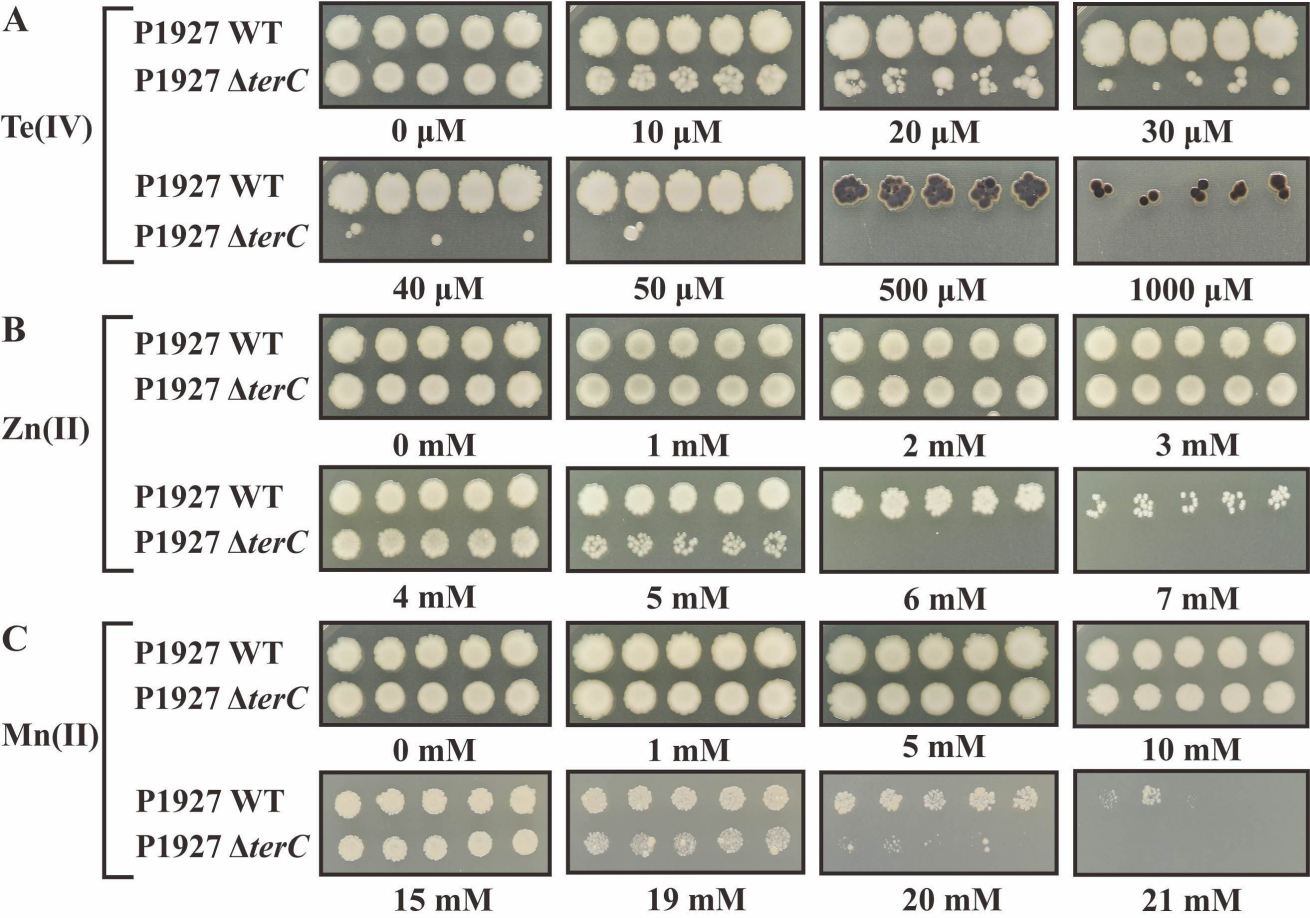
Growth phenotypes of the WT and *terC* mutant grow on the LB agar plate supplemented with a range of Te(IV) (**A**), Zn(II) (**B**), and Mn(II) (**C**) at 37°C. Data are five (*n* = 5) independent technical replicates.

**Fig 4:**
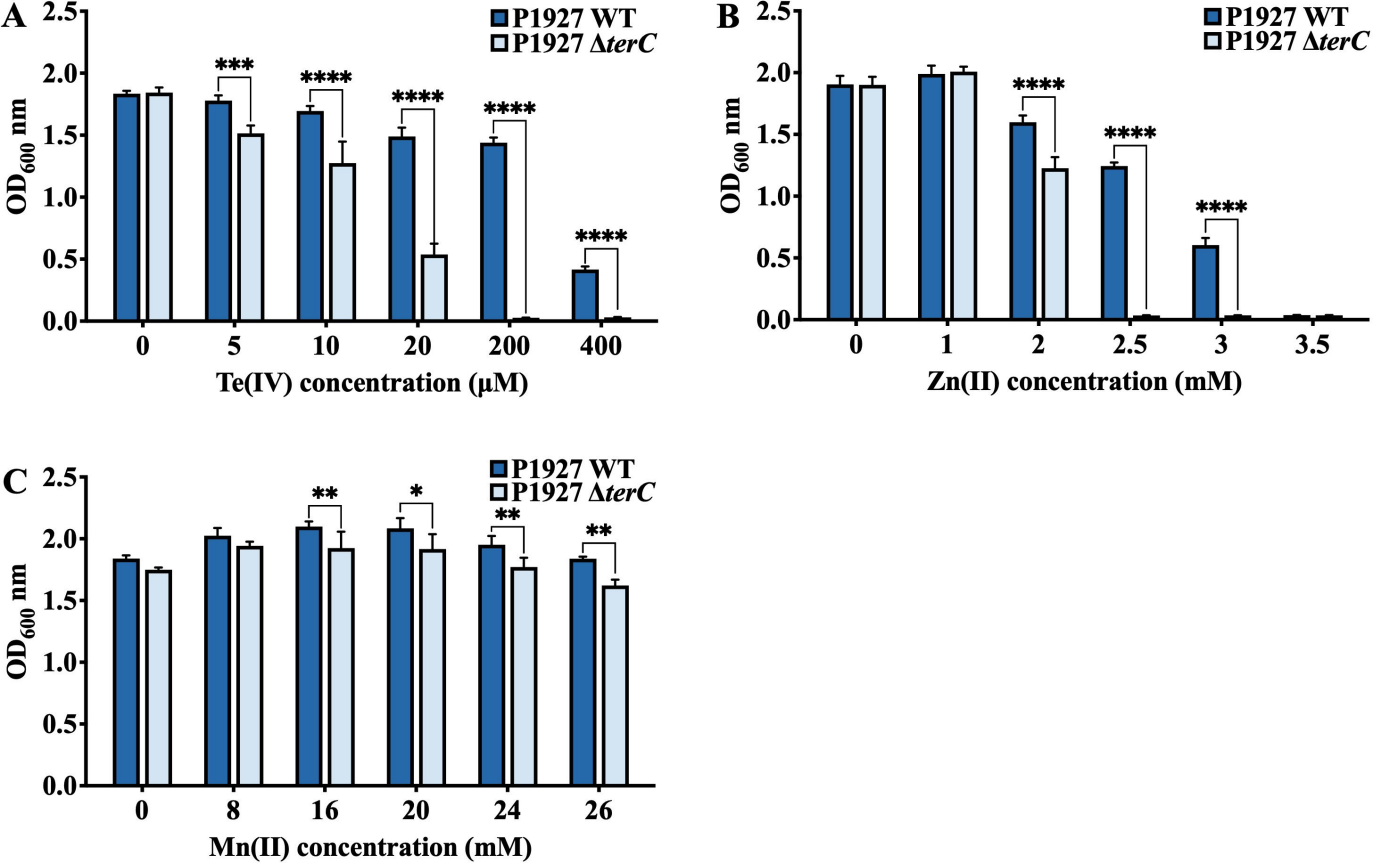
Growth phenotypes of the WT and *terC* mutant grow in LB liquid medium supplemented with a range of Te(IV) (**A**), Zn(II), (**B**) and Mn(II) (**C**) at 37°C. Data are mean OD_600 nm_ values (± SD) from three (*n* = 3) independent biological experiments. Statistical significance of the differences determined by two-way ANOVA with Sidak posttest: **** (*P* < 0.0001), *** (*P* < 0.001), ** (*P* < 0.01), and * (*P* < 0.05), using GraphPad Prism 9.5.1.

It has previously been reported that the *terCDE* genes are more conserved than the *terZAB* genes (22). Moreover, the TerC protein is one of the key proteins in conferring tellurite resistance (43) and has independently been implicated in Mn(II) detoxification (44). Therefore, to determine the function of tellurium resistance genes in *K. pneumoniae* P1927, a *terC* deletion mutant was generated. The *terC* mutant and wild-type (WT) cells were grown in the presence of various heavy metal(loid)s at a range of concentrations, and their phenotypes were compared. The *terC* mutant was only able to tolerate 30 µM supplemental Te(IV), whereas the WT grew in the presence of up to 1,000 µM Te(IV) on LB agar plate (more than 33-fold change) (Fig. 3A; Table 2). Additionally, the WT grew better than the *terC* mutant in LB liquid medium containing 5 µM Te(IV), and the WT was able to grow well in LB liquid medium supplemented with 400 µM Te(IV), whereas the *terC* mutant showed no growth under the same conditions (Fig. 4A). The WT formed black colonies on LB agar plates containing 500 µM and 1,000 µM Te(IV) (Fig. 3A), suggesting that TerC plays a pivotal role in Te(IV) reduction (45).

Surprisingly, we observed that the WT was able to grow on LB agar plates in the presence of 7 mM Zn(II) or 21 mM Mn(II) (Fig. 3B and C; Table 2), whereas the *terC* mutant was slightly more sensitive, with an upper limit of 5 mM Zn(II) (1.4-fold change) (Fig. 3B) and 20 mM Mn(II) (1.05-fold change) (Fig. 3C). In LB liquid medium, the WT was resistant up to 3 mM Zn(II), whereas the *terC* mutant showed no growth under the same conditions (Fig. 4B). In contrast, both the WT and the *terC* mutant grew well in LB liquid medium containing a range of Mn(II) concentrations, although significant differences in growth were observed in the presence of 16 mM, 20 mM, 24 mM, and 26 mM Mn(II) (Fig. 4C). Additionally, no differences in resistance to Cu(II), Ni(II), Co(II), As(III), Ag(I), Cd(II), Pb(II), Sb(III), Sb(V), or Rox(V) (Roxarsone) were observed between the WT and the *terC* mutants (Fig. S2). However, we found that Zn(II) and Mn(II) promoted the growth of both the WT and the *terC* mutant at certain concentrations (Fig. S3). These assays revealed that TerC not only contributes to tellurite resistance but also plays a role in the high levels of Zn(II) and Mn(II) resistance observed. We also tested the resistance of the WT and the *terC* mutant to hydrogen peroxide, showing the deletion of *terC* did not result in decreased resistance (Fig. S4).

### The *ter* operon was inducible by Te(IV), Zn(II), and Mn(II)

**Fig 5:**
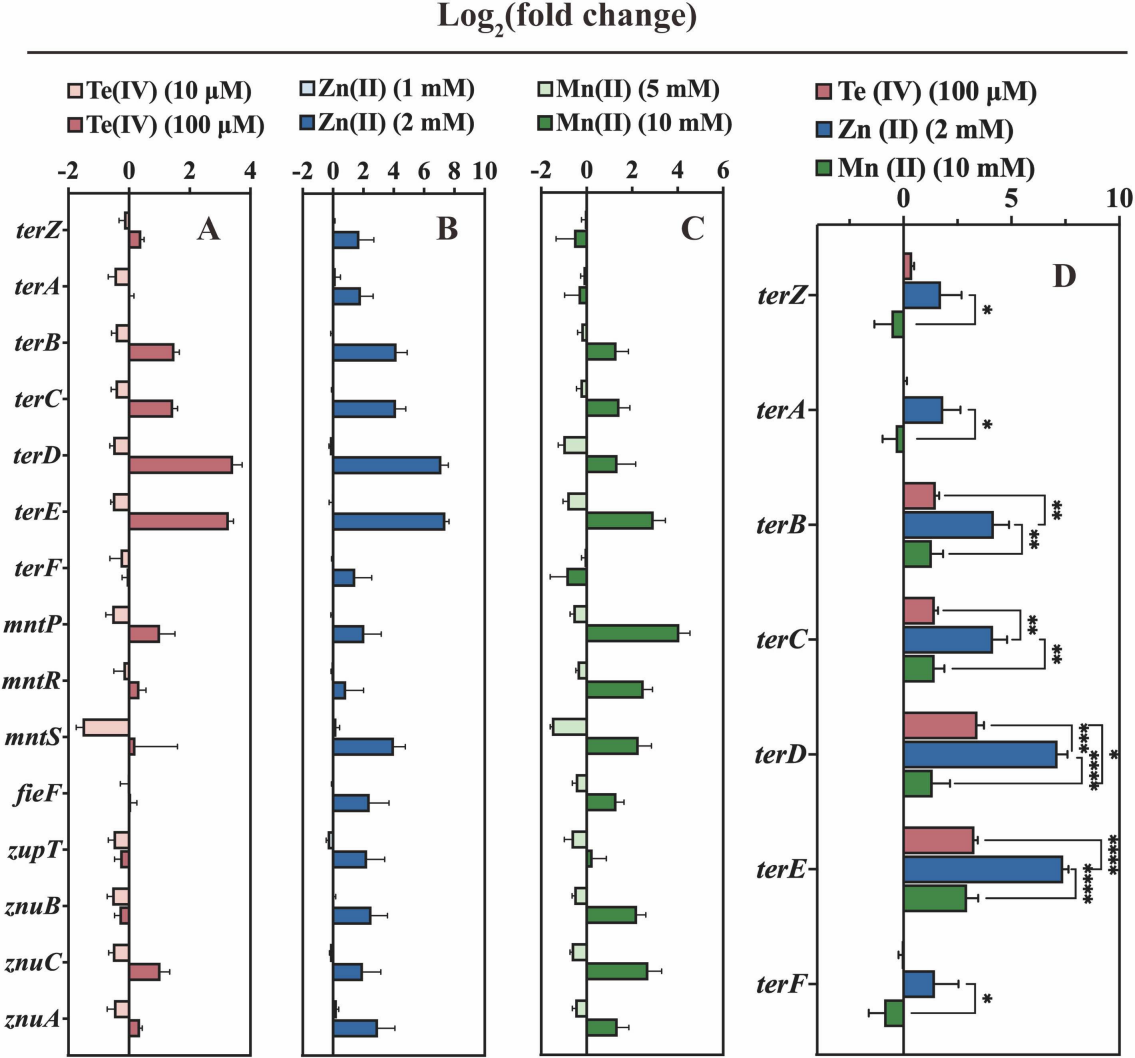
RT-qPCR analysis of tellurium resistance genes (*terZABCDEF*), manganese resistance genes (*mntPRS*), and zinc resistance genes (*fieF*, *zupT,* and *znuBCA*) from *K. pneumoniae* P1927. Strain was grown in LB liquid medium containing 10 µM and 100 µM Te(IV) (**A**), 1 mM and 2 mM Zn(II) (**B**), and 5 mM and 10 mM Mn(II) (**C**) for 1.5 h, and RNA was isolated and analyzed by RT-qPCR. (D) Expression of *ter* operon in LB liquid medium supplemented with 100 µM Te(IV), 2 mM Zn(II), and 10 mM Mn(II). Log_2_ fold change in the expression for treated *K. pneumoniae* P1927 WT cultures, by comparison with untreated. Data are mean values (± SD) from three (*n* = 3) independent biological experiments. Statistical significance of the differences determined by two-way ANOVA with Sidak posttest: **** (*P* < 0.0001), *** (*P* < 0.001), ** (*P* < 0.01), and * (*P* < 0.05), using GraphPad Prism 9.5.1.

We next performed quantitative reverse transcription-PCR (qRT-PCR) under Te(IV), Zn(II), and Mn(II) stress to measure the expression of genes encoding tellurium resistance (*terZABCDEF*), manganese resistance (*mntPRS*), and zinc resistance (*fieF*, *zupT,* and *znuBCA*) in *K. pneumoniae* P1927. FieF, also known as YiiP, has previously been shown to facilitate zinc and iron transport (46, 47) and was recently reported to contribute to manganese resistance and efflux in *Salmonella typhimurium* (48). We observed that the expression levels of the *terZBCDE*, *mntPRS*, and *znuCA* genes were upregulated in the presence of 100 µM Te(IV) but not by 10 µM Te(IV) (Fig. 5A). All tested genes were upregulated in response to 2 mM Zn(II) (Fig. 5B). The expression levels of *terBCDE*, *mntPRS*, *fieF*, *zupT*, and *znuBCA* were also upregulated by 10 mM Mn(II) (Fig. 5C). For all genes, induction by Zn(II) was significantly higher than that by other stressors (Fig. 5D).

### A deletion of *terC* exhibited decreased phage resistance

**Fig 6:**
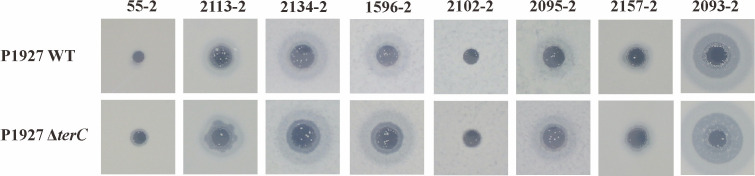
Phage plaques of phage 55–2, 2113–2, 2134–2, 1596–2, 2102–2, 2095–2, 2157–2, and 2093–2 on the LB agar plates. Figures are the same scales (250 × 250 pixels).

Transpositions into *terC*, *terD*, and *terZ* were found to reduce or abolish phenotypes related to phage inhibition, tellurite resistance, and colicin resistance in 1995 (23). Therefore, eight phages, 55–2, 2113–2, 2134–2, 1596–2, 2102–2, 2095–2, 2157–2, and 2093–2 were isolated from hospital sewage and used to assess phage resistance in the WT and the *terC* mutant. In spot assays, all eight tested phages were able to lyse both the WT and the *terC* mutant strain. However, these phages formed large clear plaques against the *terC* mutant while forming small clear plaques against the WT on LB agar plates (Fig. 6). In efficiency of plating (EOP) assays, only phage 2095–2 formed turbid zones with a high EOP on LB agar plates containing the *terC* mutant compared with the WT (Fig. S5).

**Fig 7:**
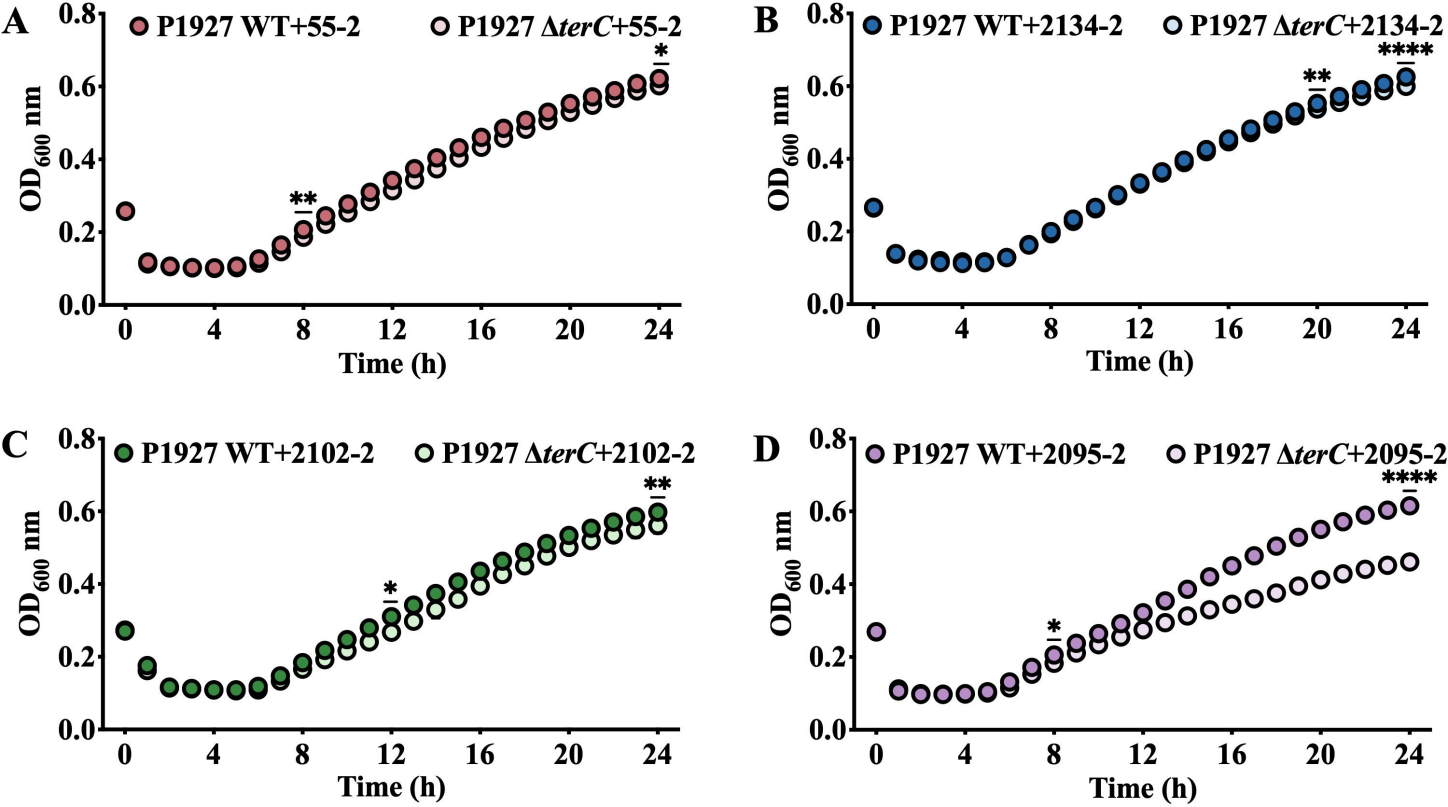
Growth of the WT and *terC* mutant in the presence of phage 55–2 (**A**), 2134–2 (**B**), 2102–2 (**C**), and 2095–2 (**D**) over 24 h in LB liquid medium. Data are mean OD_600 nm_ values (± SD) from three (*n* = 3) independent biological experiments. Statistical significance of the differences determined by two-way ANOVA with Sidak posttest: **** (*P* < 0.0001), *** (*P* < 0.001), ** (*P* < 0.01), and * (*P* < 0.05), using GraphPad Prism 9.5.1.

Furthermore, the WT and the *terC* mutant were co-cultured with each of the eight phages in LB liquid medium in 96-well microtiter plates, and growth curves were monitored at OD_600 nm_. Within 1–2 h of phage infection, the relative growth rate of the WT and the *terC* mutant in all phage-treated wells declined rapidly (Fig. 7; Fig. S6). We observed that in the presence of phages 55–2, 2134–2, 2102–2, and 2095–2, the relative growth rates of the WT and the *terC* mutant first showed significant differences at 7, 20, 11, and 8 h, respectively (Fig. 7). The relative growth of the *terC* mutant in the presence of phages 55–2, 2134–2, 2102–2, and 2095–2 was slower, and the final bacterial abundance was significantly decreased compared with the WT (Fig. 7), suggesting that deletion of *terC* decreased resistance to these phages. However, there were no significant differences in the relative growth rates between the WT and the *terC* mutant in the presence of phages 2113–2, 1596–2, 2157–2, and 2093–2 (Fig. S6).

### A deletion of *terC* decreased virulence in *K. pneumoniae* P1927

**Fig 8:**
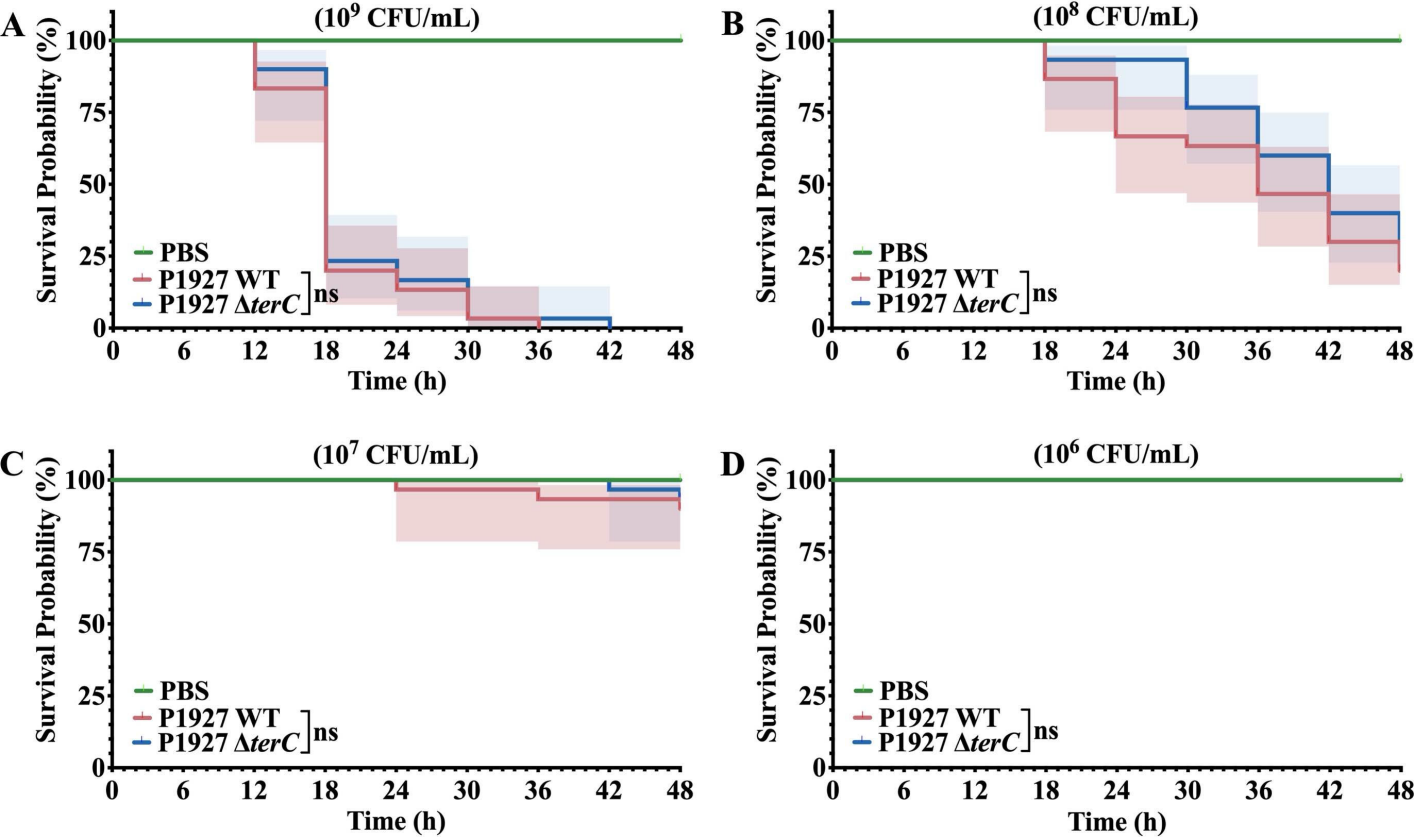
Kaplan–Meier plots showing the percent survival of *G. mellonella* larvae over 48 h post-infection with 10^9^ colony-forming units (CFU) (**A**), 10^8^ CFU (**B**), 10^7^ CFU (**C**), and 10^6^ CFU (**D**) of the WT or *terC* mutant. The experiments were controlled by PBS-injected larvae. Survival curves were plotted using the Kaplan–Meier method (GraphPad Prism 9.5.1 software). Each experiment was performed in triplicate with 10 animals per treatment per replicate, and shaded areas show 95% confidence intervals in survival probability. Statistical significance of the differences determined by the Log-rank test using GraphPad Prism 9.5.1.

The insect *Galleria mellonella* larva is a widely used model for bacterial pathogenesis, as its immune response is similar to the innate immune system of mammals in a number of structural and functional characteristics (49, 50). To determine whether the *terC* mutant had reduced virulence compared with the parent strain, larvae were infected with the WT and the *terC* mutant strains. The survival rates of larvae injected with high concentrations (10^9^ CFU) of the WT or the *terC* mutant decreased sharply, indicating that both the WT and the *terC* mutant were virulent at high concentrations (Fig. 8A). However, at a concentration of 10^8^ CFU, the *terC* mutant reduced the virulence of the strain, with increased larval survival rate compared with the larvae injected with the WT strain. However, the comparison of survival rates did not show a significant difference (Fig. 8B, log-rank test *P* = 0.4234). In contrast, both the WT and the *terC* mutants exhibited low virulence at low concentrations, and no significant difference in virulence was observed between the WT and the *terC* mutants (Fig. 8C and D). These results demonstrate a minor role of *terC* in the pathogenicity of *K. pneumoniae* P1927.

## DISCUSSION

*K. pneumoniae* constitutes a major clinical and public health threat to humans, and numerous virulence factors contributing to its pathogenicity have been identified (51, 52). In this study, we determined that *terC*, part of a tellurium resistance determinant, also functions as a virulence factor. The *ter* operon was originally named based on its association with the reduction of tellurite to black metallic tellurium (53). Our results demonstrate that deletion of *terC* not only decreased Te(IV) resistance (Fig. 3A and 4A) but also slightly reduced virulence (Fig. 8) of *K. pneumoniae* P1927 during *G. mellonella* larva infection. Coincidentally, a recent study showed that deletion of *terC* led to a tellurite-sensitive phenotype, but no significant fitness defect was observed in a pneumonia model of infection (lungs of mice) (32). Nevertheless, *terC* has been linked to the virulence of *K. pneumoniae*, and recent studies have connected the *ter* operon to fitness and gut colonization in *K. pneumoniae* (28, 32, 33). These studies suggest that the *ter* operon is a conserved fitness factor that enhances the virulence and pathogenicity of *K. pneumoniae* during infection. More alarmingly, *ter* operons have already been widely discovered among pathogenic bacterial isolates, indicating that their acquisition could improve the fitness and virulence of pathogenic bacteria, enabling them to resist environmental competitive pressures and expand their host range (14, 28, 54).

Surprisingly, we also observed that deletion of *terC* decreased not only Te(IV) resistance but also Zn(II) resistance while also slightly decreasing resistance to Mn(II) (Fig. 3B, C, 4B and C). In *B. subtilis*, TerC family proteins also play a very modest role in Mn resistance in strains that additionally carry the major Mn(II) efflux pumps, MneP and MneS (44). Indeed, the physiological role of *B. subtilis* TerC is likely related to the metalation of secreted proteins rather than detoxification (35). Similarly, the contribution of TerC in strain P1927 to Mn(II) and Zn(II) detoxification may be largely obscured by the presence of other HMR genes, and TerC may have other functions not yet defined.

The genes of the *ter* operon were highly inducible by Zn(II) and Mn(II) at high concentrations (2 mM/10 mM) (Fig. 5). Zn(II) and Mn(II) are essential for survival, colonization, and pathogenesis in the infected host (55). However, transition metals are toxic at high concentrations, and one of the host defense strategies against infection consists of using metal toxicity to kill pathogens by the highly concentrated release of various metals (56, 57). Therefore, *terC*, possibly in conjunction with other *ter* operon genes, may contribute to HMR and thereby facilitate pathogen survival in response to the host immune system. This could be one reason why deletion of *terC* decreased the virulence of *K. pneumoniae* P1927 during *G. mellonella* larva infection. In *E. coli*, the *ter* operon enhanced bacterial survival in the event of a macrophage attack (25). In *Yersinia pestis*, similarly, a *terZABCDE* deletion mutant abolished the filamentous morphologic response during macrophage infections (26). These findings suggest that the presence of the *ter* operon provides pathogens with an adaptive strategic response against the immune response of the host organism.

The mechanism underlying TerC-mediated resistance to Zn(II) and Mn(II) in *K. pneumoniae* P1927 remains unclear. The results of heterologous expression of *K. pneumoniae* P1927 TerC in a zinc-sensitive strain suggested that it is not an efflux transport protein for Zn(II)/Mn(II) (Fig. S7). Moreover, ZntA is known to be a very effective, powerful P-type ATPase that is sufficient to pump out any excess Zn from the cytoplasm. Proteomic analysis of the TerC interactome from a recent study showed that the TerC-TerB complex appears to act as a central unit that may link different functional modules with biochemical activities C4 dicarboxylate transport, inner membrane stress response, ATPase/chaperone activity, and proteosynthesis (22). These results suggest that TerC, together with TerB, forms the TerC-TerB complex, which should be a core functional protein interacting with determinants involved with stress response, fitness, and survival for the bacterial isolates, also explaining the various functions of *terC* in *K. pneumoniae* P1927. However, other recent results indicated that TerC-TerD forms the minimal functional unit with either TerZCD or TerACD conferring full resistance to tellurium in *E. coli* (43). Therefore, these findings highlight that more efforts need to be extended to better understand the interaction between TerC and other encoded proteins of the *ter* operon complex in conferring increased fitness to the pathogenic strain, Zn(II)/Mn(II) resistance, phage resistance, and other functions.

Many *K. pneumoniae* strains exhibit extensive phenotypic and genetic diversity due to the acquisition of AMR, virulence, and other accessory genes through horizontal gene transfer (58). Conjugation is one of the most important means of dissemination of AMR and virulence factors, and the encoded proteins of the *tra*/*trb* cluster are required for conjugative plasmids (59, 60). Two of the plasmids described here carried the *tra*/*trb* cluster (Fig. 1), suggesting that they could facilitate their self-transmissibility and be readily transferred in bacterial populations. Therefore, conjugation experiments should be performed in the future to investigate the transmissibility of these plasmids. Moreover, genes encoding resistance to copper, silver, mercury, nickel, cadmium, and arsenic were also found on the plasmids in *K. pneumoniae* P1927 (Fig. 1). It has been reported that the abundance and mobility of HMR genes contribute to the dissemination and maintenance of AMR genes, and many of these genes are located on transmissible plasmids (61, 62). This suggests that pathogenic bacterial isolates carrying diverse HMR determinants could be a reservoir for disseminating AMR genes, which will be transmitted between bacteria by horizontal gene transfer, generating extremely resistant pathogenic isolates and possessing a potential risk to human health by the food chain (63, 64). Additionally, zinc, manganese, and copper resistance determinants have also been shown to be critical for the virulence of pathogenic bacteria (65–70). Therefore, including the *ter* operon, HMR genes, and their association with virulence and stress tolerance highlights the need to better understand the interaction between the HMR genes and pathogenic bacteria, including core and variable physiological functions, host factors, and the role of virulence and gut colonization, which is predicted to have many benefits for informing the design of novel therapeutics and control strategies.

## MATERIALS AND METHODS

### Phages, bacterial strains, and growth conditions

Phages, strains, and plasmids used in this study are listed in Table S1. The phages and their hosts were kindly provided by the Environmental Bioelectrochemistry Center (Fujian Agricultural and Forestry University, Fuzhou, China). The parent strain, *K. pneumonia* P1927, was isolated from patient sputum at Fujian Provincial Hospital and was used to generate the *terC* mutant by homologous recombination. *K. pneumoniae* 2095, 1596, 2102, and 2134 are phage-host strains and were used for phage infection assays. *E. coli* ATCC 25922 is a quality-control strain for heavy metal(loid)s or antimicrobials resistance assay (61). *E. coli* DH5α and S17-1λpir were competent cells used for plasmid DNA construction and replication. In this study, *K. pneumoniae* strains were grown aerobically in lysogeny broth (LB) medium at 37°C with shaking at 180 rpm. *E. coli* strains were grown aerobically in LB medium supplemented with 100 µg/mL ampicillin or 50 µg/mL apramycin as required at 37°C with shaking at 180 rpm for most experiments. Bacterial growth was monitored by measuring the absorbance at OD_600 nm_.

### Genome sequencing and analysis of *K. pneumoniae* P1927

Total genomic DNA (gDNA) was extracted from *K. pneumoniae* P1927 using the TIANamp Bacteria DNA Kit (TIANGEN Biotech, Beijing, China) according to the manufacturer’s protocol. The gDNA was subsequently sequenced using the Oxford Nanopore PromethION sequencing platform. The quality of the Illumina sequencing data was assessed using FastQC v0.11.8 and Trimmomatic v0.39 for adapter clipping, quality trimming, and minimum length exclusion (>50 bp). The sequence reads were assembled using Canu v1.5 software. Gene prediction and annotation were performed using the Prokaryotic Genome Annotation Pipeline (PGAP) v.6.1 in the NCBI (71) and Prokaryotic Genome Annotation Service of v.2.0 in the Rapid Annotations using Subsystems Technology (RAST) (72). Functional annotation and antimicrobial resistance genes were checked using ResFinder (v.4.3.3) at the Center for Genomic Epidemiology (http://genepi.food.dtu.dk/resfinder) and the Comprehensive Antibiotic Resistance Database (https://card.mcmaster.ca/home) (73, 74). The multilocus sequence typing (MLST) and capsule type of the strain were determined using MLST (v.2.0) in Center for Genomic Epidemiology (https://cge.food.dtu.dk/services/MLST/) and Kaptive, respectively (75, 76). The typing of plasmids was determined using PlasmidFinder (v.2.1) at the Center for Genomic Epidemiology (https://cge.food.dtu.dk/services/PlasmidFinder/) (75). The final assembled circular chromosome and plasmids were visualized using CGView (v.1.0) (77). Unless otherwise specified, all programs were run using the default parameters. The genomic data sets were deposited in the NCBI databases under accession numbers CP073377.1, CP073378.1, CP073379.1, and CP073380.1.

### Construction of the *terC* deletion

Primers used for mutant strain construction are listed in Table S2. The *terC* deletion mutant was constructed in *K. pneumoniae* P1927 by allelic exchange using PJQ-200R6K, a suicide vector that allows the use of sucrose for counterselection (78–80). Fragments of 500–800 bp upstream and downstream of the target gene were amplified and joined by overlap PCR. The resulting product was cloned into PJQ-200R6K and verified by sequencing. The plasmid PJQ-Δ*terC* was then introduced into *E. coli* S17-1 *λpir* donor strain via CaCl_2_-mediated heat shock transformation. Overnight cultures of *K. pneumoniae* P1927 and *E. coli* S17-1 *λpir* carrying PJQ-Δ*terC* were centrifuged and washed twice with sterile 1× PBS. The cells were then mixed and plated on LB agar plates (without antimicrobials) and incubated at 37°C for 48 h. Single-crossover mutants were selected on LB agar plates supplemented with 100 µg/mL ampicillin and 50 µg/mL apramycin at 37°C for 48 h. Double-crossover mutants were selected on LB agar plates supplemented with 10% sucrose at 37°C for 48 h. The resulting colonies were screened for apramycin sensitivity. The deletion mutant was confirmed by PCR amplification across the deleted region.

### Heavy metal(loid)s/antimicrobials sensitivity assays

Antimicrobial resistance assays were performed by the agar dilution using Mueller-Hinton Agar (Solarbio, Beijing, China), as previously described (81, 82). Overnight cultures of WT and *terC* mutant strains were sub-cultured to mid-log phase (OD_600 nm_: 0.5–0.6) and diluted 100-fold using fresh LB medium. A 3 µL aliquot of the diluted cultures was spotted onto Mueller-Hinton Agar (MHA) plates containing various concentrations of antimicrobials. The plates were then incubated at 37°C for 24 h. The *E. coli* ATCC 25922 strain was used as a negative control. The MICs of strains were determined by observing cell growth on the plates (more than three single colonies at least three inoculation sites).

Heavy metal(loid)s resistance assays were also performed by the agar dilution using Mueller-Hinton Agar (Solarbio, Beijing, China), as previously described (83). Overnight cultures of WT and *terC* mutant strains were adjusted to an OD_600 nm_ value of 0.5–0.6 and then diluted 100-fold using LB medium. A 3 µL aliquot of the adjusted bacterial cultures was spotted onto MHA plates containing various concentrations of heavy metal(loid)s. The plates were then incubated at 37°C for 24 h. For metal(loid)s resistance assays in liquid media, *K. pneumoniae* P1927 and *terC* mutant strains were grown overnight in LB medium with shaking at 37°C. The overnight cultures were diluted 100-fold in fresh LB medium containing various concentrations of metal(loid)s and incubated at 37°C with shaking for 24 h. The growth conditions were estimated using the absorbance at OD_600 nm_. Experiments were performed with three independent biological replicates.

### Quantitative real-time PCR

A single colony of *K. pneumoniae* P1927 WT was incubated in LB medium overnight at 37°C with shaking at 180 rpm. Overnight cultures were sub-cultured in LB medium until reaching mid-log phase (OD_600 nm_: 0.5–0.6). Then, 10/100 µM Te(IV), 1/2 mM Zn(II), and 5/10 mM Mn(II) were added, respectively. Each treatment was performed with three independent biological replicates. The treatment without metal addition was used as a control. Research-grade chemicals, K_2_O_3_Te, ZnSO_4_·7H_2_O, and MnSO_4_ were used for Te(IV), Zn(II), and Mn(II), respectively. After incubation for 1.5 h, bacterial suspensions were harvested by centrifugation at 12,000 rpm for 2 min. Total RNA was extracted using the *TransZol* UP Plus RNA kit (TransGen Biotech, Beijing, China) according to the manufacturer’s instructions. RNA concentrations were quantified using a NanoDrop 2000 Microvolume Spectrophotometer (Thermo Fisher Scientific, Waltham, USA). The RNA was then diluted to appropriate concentrations (15 µL, about 40 ng/µL). Complementary DNA (cDNA) synthesis with DNA integrated genomic DNA (gDNA) removal was performed using *TransScript* Uni All-in-One First-Stand cDNA Synthesis SuperMix for qPCR (One-Step gDNA Removal) kit (TransGen Biotech, Beijing, China). Quantitative real-time PCR was performed using the *TransStart* Tip Green qPCR SuperMix kit (TransGen Biotech, Beijing, China) on a QuantStudio 6 Flex real-time PCR system (Thermo Fisher Scientific, Waltham, USA). The resulting cDNA was used as the template. The reactions (20 µL) containing forward primer (0.4 µL, 10 µM), reverse primer (0.4 µL, 10 µM), 2× *PerfectStart* Green qPCR SuperMix (TransGen Biotech, Beijing, China) (10 µL), cDNA template (1.2 µL), and nuclease-free water (8 µL). The 16S rRNA of *K. pneumoniae* P1927 WT was used as an endogenous control. Relative expression results were obtained by the ΔΔCT analysis method using mean CT value, as previously described (84). Primers for quantitative real-time PCR were designed using Primer3plus (https://www.primer3plus.com) and are listed in Table S2.

### Propagation of bacteriophage

Proliferation and purification of phages were performed using the double-layer agar method as previously described (85). Phages and their host strains were co-cultured in LB medium at 37°C with shaking at 180 rpm for 5–8 h. The cultures were harvested by centrifugation at 12,000 rpm for 3 min. The supernatant was then serially diluted 10-fold (10–10^9^) using LB medium. A 100 µL aliquot of the diluted supernatant was mixed with 100 µL of phage-host strain culture (OD_600 nm_: 0.3) and incubated at 37°C for 10 min. A 4.8 mL volume of semi-solid LB medium (0.8% agar) was added to the mixture and poured onto the LB agar plates (1.5% agar). The plates were incubated at 37°C overnight. Plaques were collected in the tube and stored using 1× SM buffer (Sangon Biotech, Shanghai, China) at 4°C overnight to release phage particles. The supernatant was filtered through a 0.22 µm syringe filter (Jinteng, Tianjin, China) and stored at 4°C until further use.

### Susceptibility of *K. pneumoniae* P1927 and *terC* deletion mutant to phage infection

Phage titer was determined as described above. Overnight cultures of the WT and the *terC* mutant strains were sub-cultured to mid-log phase (OD_600 nm_, 0.3). The phage solution was serially diluted 10-fold (10-10^9^) using LB medium. A 100 µL aliquot of the diluted phage solution was mixed with 100 µL of the WT/*terC* mutant strain culture (OD_600 nm_, 0.3) and incubated at 37°C for 10 min. A 4.8 mL volume of semi-solid LB medium (0.8% agar) was added to the mixture and poured onto LB agar plates (1.5% agar). The plates were incubated at 37°C for 24–48 h. After incubation, plaques were counted to determine the phage titer. Each treatment was performed with three independent biological replicates.

Bacterial growth curves for phage resistance assays in LB were generated. To assess the growth of the WT/*terC* mutant strain in the presence of phages, overnight cultures were sub-cultured to mid-log phase (OD_600 nm_, 0.3). A 180 µL aliquot of the culture was mixed with 20 µL of phage solution. The mixture was then added to 96-well microtiter plates. The mixture was incubated at 37°C and measured at OD_600 nm_ every 15 min for 24 h under aerobic shaking conditions using a BioTek Cytation 5 plate reader (Gen5 v3.10). Each treatment was performed with three independent biological replicates.

### *G. mellonella* infection assay

*G. mellonella* infection assays were performed as previously described (86, 87). Research-grade *G. mellonella* larvae at their final instar stage were obtained in bulk from Keyun Biology (Henan, China). The larvae were stored at 15°C without food for up to 3 days before use. The health condition of the larvae was evaluated based on the following three criteria: 0.20–0.30 g weight, movement in response to touch, and absence of melanization. For survival analyses, the WT and the *terC* mutant strains were grown to a late exponential phase in LB medium and harvested by centrifugation. Bacterial pellets were washed twice with sterile 1× PBS and diluted to an appropriate cell density prior to inoculation into larvae. A 10 µL dose of diluted bacterial suspensions (10^5^–10^9^ CFU/mL) was injected into the rear left pro-leg of each larva using a 10 µL Hamilton syringe (Shanghai, China). The negative control was inoculated with 1× PBS. A group of 10 larvae were randomly selected for injection. Each treatment was performed with three independent biological groups. The infected larvae were incubated in the dark without food at 37°C for up to 48 h. Larvae were examined every 6 h, and larvae death was recorded when they were unresponsive to touch. The results were analyzed and visualized by Kaplan–Meier survival curves (GraphPad Prism statistics software).

## Data Availability

The complete genome data of K. pneumoniae P1927 has been submitted to the NCBI with accession numbers CP073377.1, CP073378.1, CP073379.1, and CP073380.1.

## SUPPLEMENTAL MATERIAL

Supplemental material spectrum.02634-24-s0001.pdf Fig. S1-S7; Table S1, S2, and S3.

ASM does not own the copyrights to Supplemental Material that may be linked to, or accessed through, an article. The authors have granted ASM a non-exclusive, world-wide license to publish the Supplemental Material files. Please contact the corresponding author directly for reuse.
