# Supplementary material for: The Klebsiella pneumoniae tellurium resistance gene terC contributes to both tellurite and zinc resistance
Source: Microbiol Spectr. 2025 Apr 9;13(5):e02634-24. doi: 10.1128/spectrum.02634-24 (PMC12054061; doi:10.1128/spectrum.02634-24)
Supplement: Supplemental material — Fig. S1-S7; Table S1, S2, and S3. [file spectrum.02634-24-s0001.pdf]

A *Klebsiella pneumoniae* tellurium resistance gene *terC* contributes to both tellurite and zinc resistance

Ruixiang Yang [a], Shuang Han [b], Yanshuang Yu [a,b], Hongru Li [c,d], John D. Helmann[e], Katharina Schaufler[f], Michael DL Johnson[g], QiuE Yang [b], Christopher Rensing [a*]

[a]Institute of Environmental Microbiology, College of Resources and Environment, Fujian Agriculture and Forestry University, Fuzhou, Fujian 350002, China.

[b]Fujian Provincial Key Laboratory of Soil Environmental Health and Regulation, College of Resources and Environment, Fujian Agriculture and Forestry University, Fuzhou, Fujian, 350002, China.

[c]Fujian Provincial Key Laboratory of Medical Big Data Engineering, Fujian Provincial Hospital, Shengli Clinical College of Fujian Medical University, Fuzhou, Fujian 350001, China.

[d]Department of Respiratory and Critical Care Medicine, Fujian Shengli Medical College, Fujian Medical University, Fujian Provincial Hospital, Fuzhou, China.

[e]Department of Microbiology, Cornell University, Ithaca, NY, 14853-8101, USA

[f]Department of Epidemiology and Ecology of Antimicrobial Resistance, Helmholtz Institute for One Health, Helmholtz Centre for Infection Research HZI, Greifswald, Germany.

[g]Department of Immunobiology, University of Arizona College of Medicine – Tucson, Tucson, Arizona, USA

***Corresponding author:**

rensing@iue.ac.cn (Christopher Rensing)

Summary

Figure S1. The plasmid replicons of the strain P1927 identified by PlasmidFinder. A, plasmid 1; B, plasmid 2; C, plasmid 3.

Figure S2. Growth phenotypes of the WT and the *terC* mutant grown on LB agar plates supplemented with a range of heavy metal(loid)s at 37 °C.

Figure S3. Growth phenotypes of the WT and the *terC* mutant grown in LB liquid medium supplemented with a range of Mn(II) at 37 °C.

Figure S4. Growth phenotypes of the WT and the *terC* mutant grown in LB liquid medium supplemented with a range of H_2O_2 at 37 °C.

Figure S5. Efficiency of plating (EOP) of phage 55-2, 2113-2, 2134-2, 1596-2, 2102-2, 2095-2, 2157-2 and 2093-2.

Figure S6. Growth of the WT and the *terC* mutant in the presence of phage 2113-2 (A), 1596-2 (B), 2157-2 (C) and 2093-2 (D) over 24 hours in LB liquid medium at 37 °C.

Figure S7. Growth of a zinc sensitive strain (*E. coli* GG252, Δ*zntA zitB yiiP*) containing plasmid pTOPO or pTOPO-*terC* in LB liquid medium at 37 °C.

Table S1. Phages, strains and plasmids used in this study.

Table S2. Primers used in this study.

Table S3. The antimicrobial resistance genes in the genome of the strain P1927 were identified based on ResFinder.

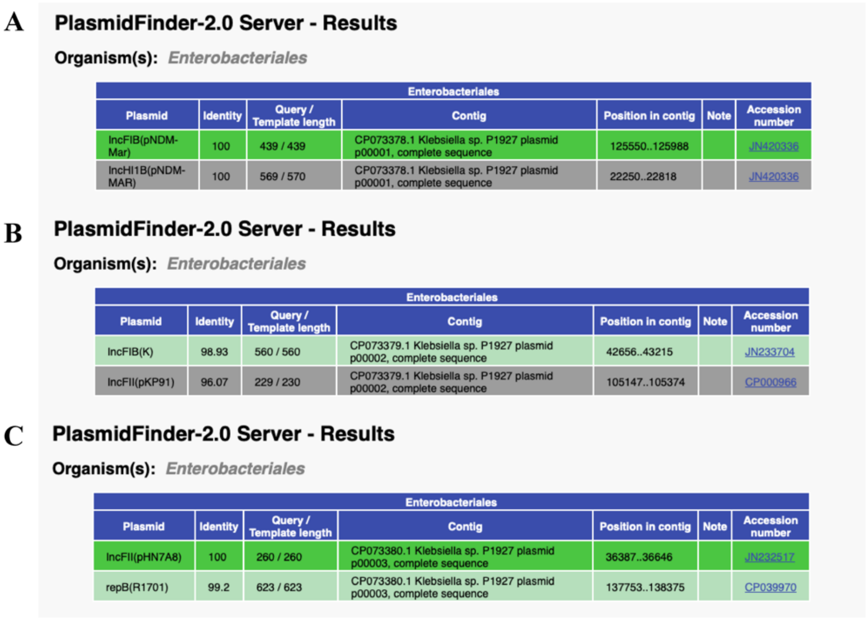

A **PlasmidFinder-2.0 Server - Results**

Organism(s): *Enterobacteriales*

Enterobacteriales						
Plasmid	Identity	Query / Template length	Contig	Position in contig	Note	Accession number
IncFIB(pNDM-Mar)	100	439 / 439	CP073378.1 Klebsiella sp. P1927 plasmid p00001, complete sequence	125550..125988		JN420336
IncHI1B(pNDM-MAR)	100	569 / 570	CP073378.1 Klebsiella sp. P1927 plasmid p00001, complete sequence	22250..22818		JN420336

B **PlasmidFinder-2.0 Server - Results**

Organism(s): *Enterobacteriales*

Enterobacteriales						
Plasmid	Identity	Query / Template length	Contig	Position in contig	Note	Accession number
IncFIB(K)	98.93	560 / 560	CP073379.1 Klebsiella sp. P1927 plasmid p00002, complete sequence	42656..43215		JN233704
IncFII(pKP91)	96.07	229 / 230	CP073379.1 Klebsiella sp. P1927 plasmid p00002, complete sequence	105147..105374		CP000966

C **PlasmidFinder-2.0 Server - Results**

Organism(s): *Enterobacteriales*

Enterobacteriales						
Plasmid	Identity	Query / Template length	Contig	Position in contig	Note	Accession number
IncFII(pHN7A8)	100	260 / 260	CP073380.1 Klebsiella sp. P1927 plasmid p00003, complete sequence	36387..36646		JN232517
repB(R1701)	99.2	623 / 623	CP073380.1 Klebsiella sp. P1927 plasmid p00003, complete sequence	137753..138375		CP039970

Figure S1. The plasmid replicons of the strain P1927 identified by PlasmidFinder. A, plasmid 1 harbored IncFIB and IncHI1B replicons; B, plasmid 2 harbored IncFIB and IncHII replicons; C, plasmid 3 harbored IncFII and repB replicons.

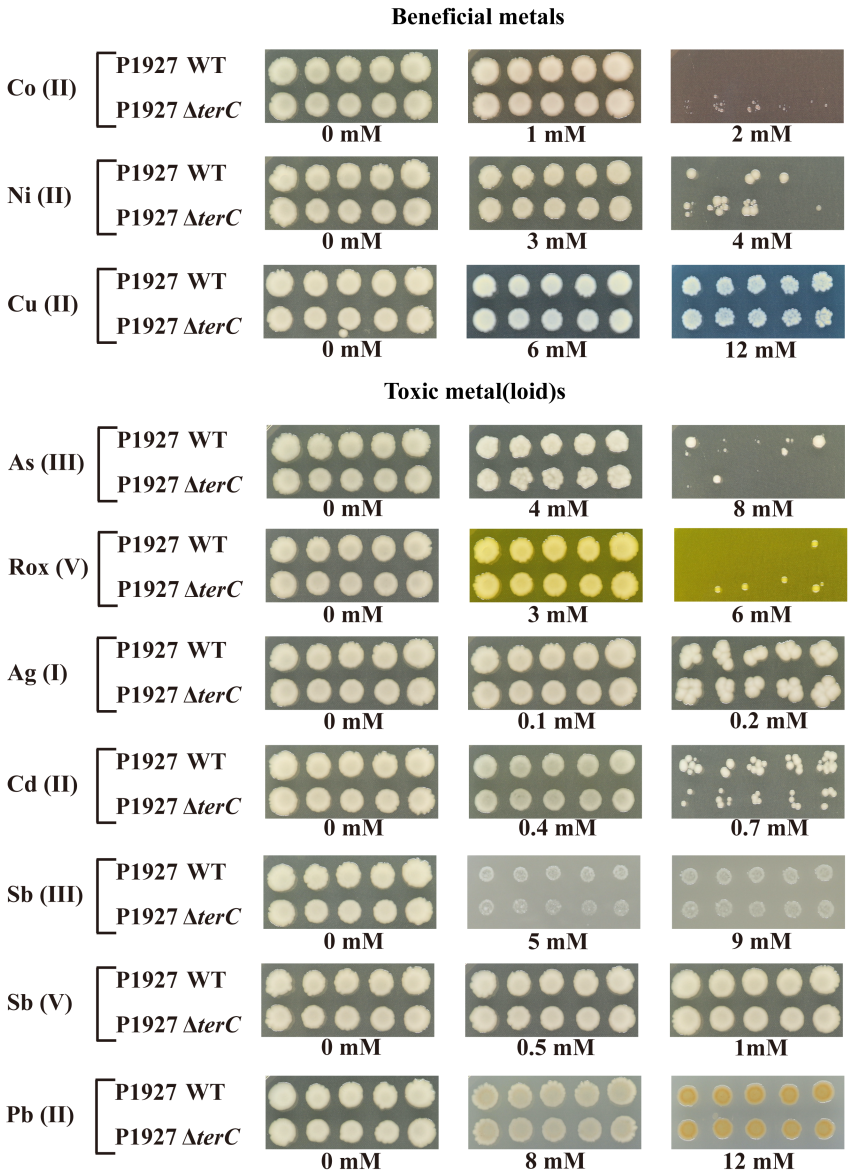

Figure S2. Growth phenotypes of the WT and the *terC* mutant grown on LB agar plates supplemented with a range of heavy metal(loid)s at 37 °C. Experiments were performed with five (n=5) independent technical replicates.

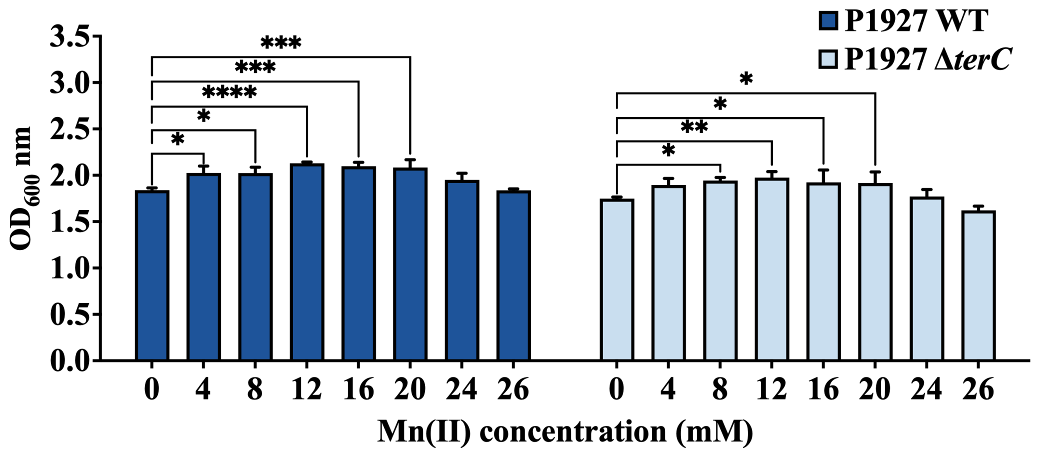

Figure S3. Growth phenotypes of the WT and the *terC* mutant grown in LB liquid medium supplemented with a range of Mn(II) at 37 °C. Data are mean $OD_{600\ nm}$ values (± SD) from three (n=3) independent biological experiments. Statistical significance of the differences determined by two-way ANOVA with Sidak posttest: **** ($p<0.0001$), *** ($p<0.001$), ** ($p<0.01$) and * ($p<0.05$), using GraphPad Prism 9.5.1.

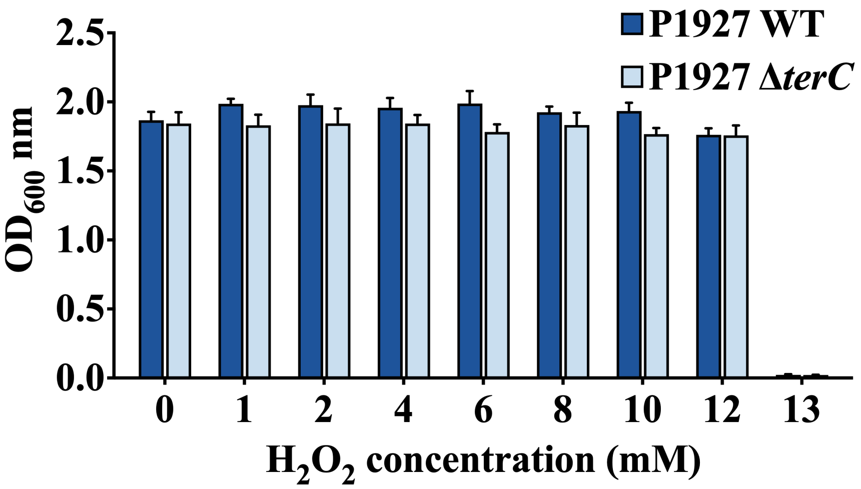

Figure S4. Growth phenotypes of the WT and the *terC* mutant grown in LB liquid medium supplemented with a range of H_2O_2 at 37 °C. Data are mean $OD_{600\ nm}$ values (± SD) from three (n=3) independent biological experiments. Statistical significance of the differences determined by two-way ANOVA with Sidak posttest: **** ($p<0.0001$), *** ($p<0.001$), ** ($p<0.01$) and * ($p<0.05$), using GraphPad Prism 9.5.1.

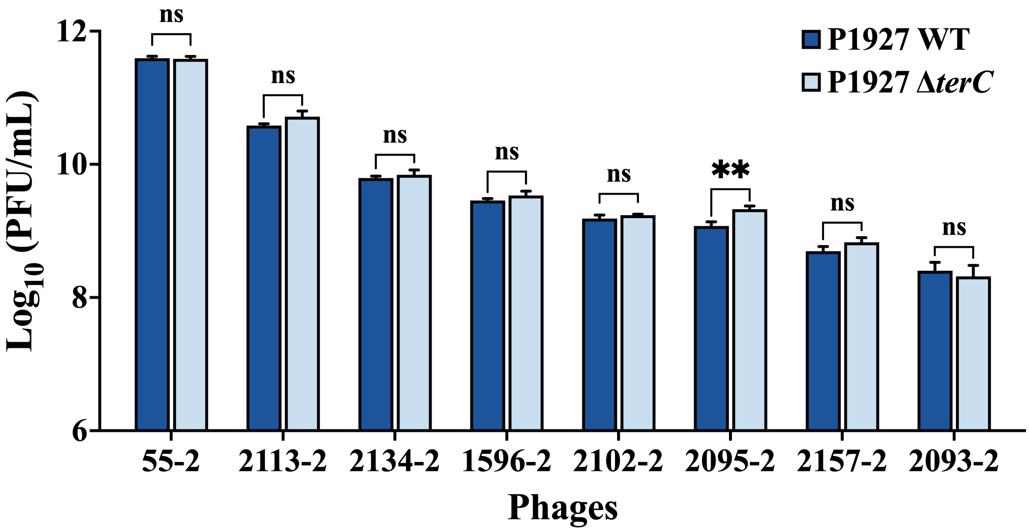

Figure S5. Efficiency of plating (EOP) of phage 55-2, 2113-2, 2134-2, 1596-2, 2102-2, 2095-2, 2157-2 and 2093-2. Data are mean Log_{10}(PFU/mL) values (± SD) from three (n=3) independent biological experiments. Statistical significance of the differences determined by two-way ANOVA with Sidak posttest: **** ($p<0.0001$), *** ($p<0.001$), ** ($p<0.01$) and * ($p<0.05$), using GraphPad Prism 9.5.1.

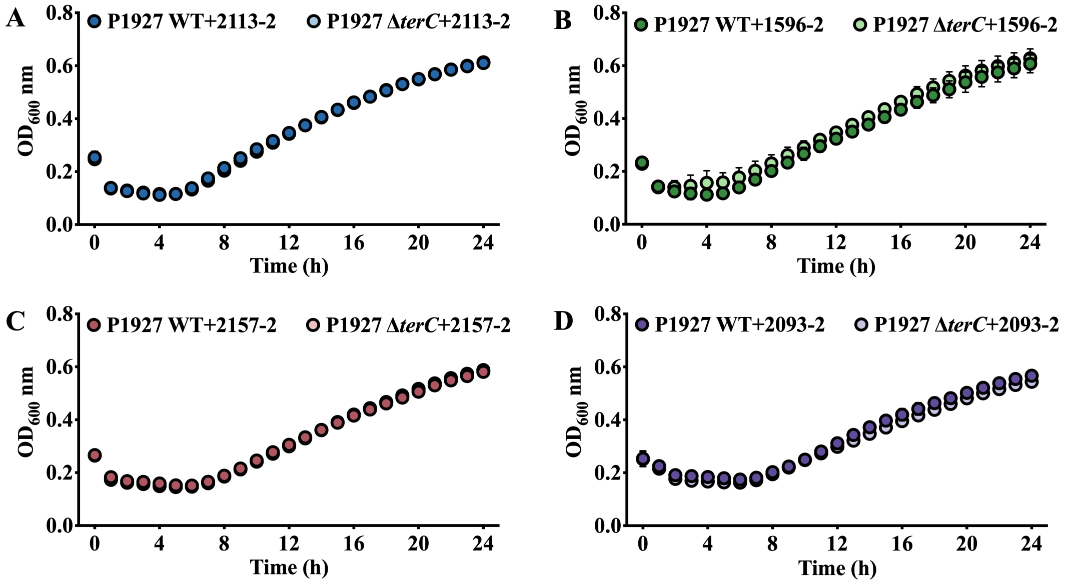

Figure S6. Growth of the WT and the *terC* mutant in the presence of phage 2113-2 (A), 1596-2 (B), 2157-2 (C) and 2093-2 (D) over 24 hours in LB liquid medium at 37 °C. Data are mean $OD_{600\ nm}$ values (± SD) from three (n=3) independent biological experiments. Statistical significance of the differences determined by two-way ANOVA with Sidak posttest: **** (p<0.0001), *** (p<0.001), ** (p<0.01) and * (p<0.05), using GraphPad Prism 9.5.1.

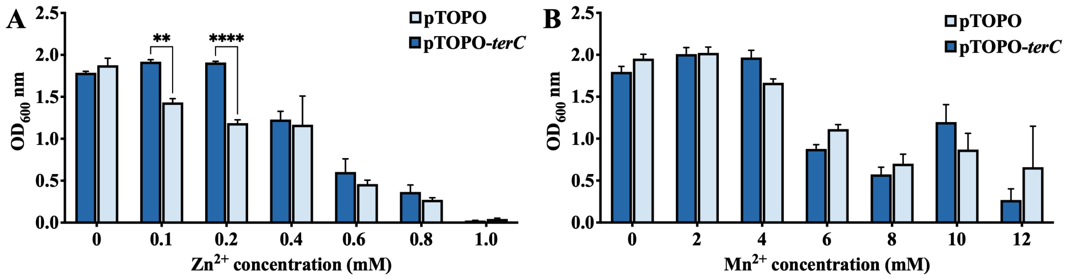

Figure S7. Growth of a zinc sensitive strain (*E. coli* GG252, Δ*zntA zitB yiiP*) containing plasmid pTOPO or pTOPO-*terC* was measured after the addition of different concentrations of Zn(II) (**A**) and Mn(II) (**B**) in liquid LB medium at 37 °C. Data are mean $OD_{600\ nm}$ values (± SD) from three (n=3) independent biological experiments. Statistical significance of the differences determined by two-way ANOVA with Sidak posttest: **** (p<0.0001), *** (p<0.001), ** (p<0.01) and * (p<0.05), using GraphPad Prism 9.5.1.

Table S1. Phages, strains and plasmids used in this study.

Phage, strain or plasmid	Characteries	Source or reference
**Phages**		
1596-2	Phage	Environmental Bioelectrochemistry Center
2095-2	Phage	Environmental Bioelectrochemistry Center
2134-2	Phage	Environmental Bioelectrochemistry Center
2102-2	Phage	Environmental Bioelectrochemistry Center
55-2	Phage	Environmental Bioelectrochemistry Center
2113-2	Phage	Environmental Bioelectrochemistry Center
2157-2	Phage	Environmental Bioelectrochemistry Center
2093-2	Phage	Environmental Bioelectrochemistry Center
**Strains**		
E. coli DH5α	Competent cell, F-φ80 lac ZΔM15 Δ(lacZYA-arg F) U169 endA1 recA1 hsdR17(rk-,mk+) supE44λ- thi -1 gyrA96 relA1 phoA	Invitrogen
E. coli GG252	zinc sensitive strain, Δ*zntA zitB yiiP*	This study
E. coli S17-1λpir	Competent cell, RP4-2(Km::Tn7, Tc::Mu-1), pro-82, LAMpir, recA1, endA1, thiE1, hsdR17, creC510	Invitrogen
E. coli ATCC 25922	Quality-control strain	This study
K. pneumonia P1927	Wilde-type, *K. pneumonia* from patient sputum	This study
K. pneumonia P1927Δ*terC*	*K. pneumonia* P1927 *terC* mutant	This study
K. pneumonia 2095	Phage host	Environmental Bioelectrochemistry Center
K. pneumonia 1596	Phage host	Environmental Bioelectrochemistry Center
K. pneumonia 2102	Phage host	Environmental Bioelectrochemistry Center
K. pneumonia 2134	Phage host	Environmental Bioelectrochemistry Center

**Plasmids**		
PJQ-R6K	Apr^r, *ori*R6K clone and expression vector	This study
PJQ-Δ*terC*	Apr^r, PJQ-R6K containing TerC heterologous region	This study

Table S2. Primers used in this study.

Primer	Sequence (5’-3’)	Use
ori R6K-F (SspI-HF)	CCCCAATATTCCCATGTCAGCCGTTAAGTG	Cloning for *ori*R6K
ori R6K-R (BmtI-HF)	CATCAGCTAGCAAGATCCGCAGTTCAACCT	
ori R6K YZ-F	CTGAAACTCTGGCTCACCGAC	Verification for *ori*R6K
ori R6K YZ-R	CTCCTGTTCAGCTACTGACGG	
TerC Up-F (SphI)	CATCGCATGCTCGGGTCGTGAGGAATTGAC	Cloning for upstream homology arm of *terC* knock out
TerC Up-R	GCTGCGCTTTTGAGGGAAATTGGCTTGTCATGTCGGTGCATG	
TerC Dn-F	AATTTCCCTCAAAAGCGCAGCCGATCACATCTGGCACCATGG	Cloning for downstream homology arm of *terC* knock out
TerC Dn-R (ApaLI)	CATCGTGCACACCAGACGGATAAATGCGCC	
YZ-F	CTGGTCAACGAGAACGGTTCC	Verification for Δ*terC*
YZ-R	CGATACAGCTCCCCAAACAGC	
YZ-F2	GAGGTTGCCAGCCTGTTTGTC	Verification for Δ*terC*
YZ-R2	CTCCAGCTGGGACAGATACTG	
PJQ-F	GCCGTGATCGAAATCCAGACC	Verification for *terC* knock out vector
PJQ-R	GCCAAGCTGTTCTTCTACGGC	
16S-F	ATTTGGGATGAAGATAGCGGTTTTC	RT-qPCR
16S-R	CAGCTTAAAGGGCTAATCAACGG	RT-qPCR
TerZ-F	TTCGTAAACTGAAATCCAGCTGTG	RT-qPCR
TerZ-R	TGTTCACAGTAAAAGCGAGGTATTC	RT-qPCR
TerA-F	TTTTGGTAACTATCACTCTGAGCCA	RT-qPCR
TerA-R	AATCAGTACCTGGCGAATTTCTTTC	RT-qPCR
TerB-F	GGTAGGCCGTTACAAAAACAAGAA	RT-qPCR
TerB-R	ATCATCTTCTGTTTTTCTTCGGAGC	RT-qPCR

TerC-F	GGTAGGCCGTTACAAAAACAAGAA	RT-qPCR
TerC-R	ATCATCTTCTGTTTTTCTTCGGAGC	RT-qPCR
TerD-F	GACGATGAATCCCTCAAAATCAAAC	RT-qPCR
TerD-R	TGTCATCATTCACCAGACGGATAA	RT-qPCR
TerE-F	GGCGGTAATGTTTCTCTGACTAAAG	RT-qPCR
TerE-R	AGGAATACTGATGCATCCAGATCAA	RT-qPCR
TerF-F	GGTGATAGATGGCAGTGATACGATA	RT-qPCR
TerF-R	CTTCCCCCATAATTACAGCCTTTTC	RT-qPCR
MntP-F	AATAAAGGCAATCCAGTGATTCCAC	RT-qPCR
MntP-R	GATTTTTGGCGCCATTGAAACC	RT-qPCR
MntR-F	GTTTGGGTAAATTTCAGGAACATCG	RT-qPCR
MntR-R	CATCAAATCGTGGAGAACTTCCTG	RT-qPCR
MntS-F	GAATTCAAGAGGTGCATAAACGTG	RT-qPCR
MntS-R	TTGATCATCTCGCACAGCATAC	RT-qPCR
ZupT-F	AACGAAATCAATAGCATAATCCCGG	RT-qPCR
ZupT-R	CAACCTTCATTGGCGCCATATT	RT-qPCR
ZnuB-F	TTCGTCATGCTGAGAATAAACAGC	RT-qPCR
ZnuB-R	ACCTTCTCGGCTTTCTATGATACC	RT-qPCR
ZnuC-F	GTTAAGATTTTGCCTGGCGTGA	RT-qPCR
ZnuC-R	CAAACCTTGTAACGCTGGAAAATG	RT-qPCR
ZnuA-F	CTTTGAAAAACACTACGGTTTGACC	RT-qPCR
ZnuA-R	CCAGTTGTGTTCTGATTTCGTGTAA	RT-qPCR

Table S3. The antimicrobial resistance genes in the genome of the strain P1927 were identified based on ResFinder.

Resistance gene	Identity	Phenotype	Notes
**Chromosome**			
aadA2b	99.87%	['streptomycin', 'spectinomycin']	
blaSHV-182	99.88%	['unknown beta-lactam']	Class A
fosA6	98.81%	['fosfomycin']	Natural in *K. pneumoniae*
**Plasmid 1**			
aac(3)-IId	99.88%	['gentamicin', 'tobramycin', 'dibekacin', 'netilmicin', 'apramycin', 'sisomicin']	
armA	100.00%	['gentamicin', 'tobramycin', 'amikacin', 'isepamicin', 'netilmicin']	
blaDHA-1	100.00%	['amoxicillin', 'amoxicillin+clavulanic acid', 'ampicillin', 'ampicillin+clavulanic acid', 'cefotaxime', 'cefoxitin', 'ceftazidime', 'piperacillin', 'piperacillin+tazobactam', 'ticarcillin', 'ticarcillin+clavulanic acid']	Class C;DHA-1-like;;Alternative name blaMOR-1;
blaSHV-12	100.00%	['amoxicillin', 'ampicillin', 'cefepime', 'cefotaxime', 'ceftazidime', 'piperacillin', 'aztreonam', 'ticarcillin', 'ceftriaxone']	Class A
blaTEM-1B	100.00%	['amoxicillin', 'ampicillin', 'piperacillin', 'ticarcillin', 'cephalothin']	Class A
msr(E)	100.00%	['erythromycin', 'azithromycin', 'quinupristin', 'pristinamycin ia', 'virginiamycin s']	ABC transporter
mph(A)	100.00%	['erythromycin', 'azithromycin', 'spiramycin', 'telithromycin']	Macrolide phosphotransferase
mph(E)	100.00%	['erythromycin']	Macrolide phosphotransferase
qnrB4	100.00%	['ciprofloxacin']	
sul1	100.00%	['sulfamethoxazole']	
**Plasmid 2**			
blaCTX-M-14	100.00%	['amoxicillin', 'ampicillin', 'cefepime', 'cefotaxime', 'ceftazidime',	Class A;Alternative name blaCTX-

		'piperacillin', 'aztreonam', 'ticarcillin', 'ceftriaxone']	M-14a, blaCTX-M-18
blaLAP-2	100.00%	['amoxicillin', 'ampicillin', 'piperacillin', 'ticarcillin', 'cephalotin']	Class A
qnrS1	100.00%	['ciprofloxacin']	
sul1	100.00%	['sulfamethoxazole']	
tet(A)	100.00%	['tetracycline', 'doxycycline']	
dfrA1	100.00%	['trimethoprim']	
**Plasmid 3**			
rmtB	100.00%	['gentamicin', 'tobramycin', 'amikacin', 'isepamicin', 'kanamycin', 'sisomicin', 'arbekacin']	
blaKPC-2	100.00%	['amoxicillin', 'amoxicillin+clavulanic acid', 'ampicillin', 'ampicillin+clavulanic acid', 'cefepime', 'cefotaxime', 'cefoxitin', 'ceftazidime', 'ertapenem', 'imipenem', 'meropenem', 'piperacillin', 'piperacillin+tazobactam', 'aztreonam', 'ticarcillin', 'ticarcillin+clavulanic acid']	Class A;Group 2f;Alternative name blaKPC-1
blaCTX-M-65	100.00%	['amoxicillin', 'ampicillin', 'cefepime', 'cefotaxime', 'ceftazidime', 'piperacillin', 'aztreonam', 'ticarcillin', 'ceftriaxone']	Class A
blaTEM-1B	100.00%	['amoxicillin', 'ampicillin', 'piperacillin', 'ticarcillin', 'cephalothin']	Class A
catA2	96.11%	['chloramphenicol']	
catA2	96.11%	['chloramphenicol']	Chloramphenicol acetyltransferase
